# Controlling the Emission Spectrum of Binary Emitting Polymer Hybrids by a Systematic Doping Strategy via Förster Resonance Energy Transfer for White Emission

**DOI:** 10.3390/mi12111371

**Published:** 2021-11-08

**Authors:** Bandar Ali Al-Asbahi, Mohamad S. AlSalhi, Amanullah Fatehmulla, Mohammad Hafizuddin Hj. Jumali, Saif M. H. Qaid, Wafa Musa Mujamammi, Hamid M. Ghaithan

**Affiliations:** 1 Department of Physics & Astronomy, College of Sciences, King Saud University, Riyadh 11451, Saudi Arabia; malsalhi@ksu.edu.sa (M.S.A.); aman@ksu.edu.sa (A.F.); sqaid@ksu.edu.sa (S.M.H.Q.); walmujammi@ksu.edu.sa (W.M.M.); 436107632@student.ksu.edu.sa (H.M.G.); 2 Department of Physics, Faculty of Science, Sana’a University, Sanaa 12544, Yemen; 3 Laser Diagnosis of Cancers, College of Sciences, King Saud University, Riyadh 11451, Saudi Arabia; 4 School of Applied Physics, Faculty of Science and Technology, Universiti Kebangsaan Malaysia, Bangi 43600, Selangor, Malaysia; hafizhj@ukm.edu.my; 5 Department of Physics, Faculty of Science, IBB University, Lapai 70270, Yemen

**Keywords:** binary hybrids, Förster resonance energy transfer, conjugated polymers, white emission

## Abstract

Tuning the emission spectrum of both binary hybrids of poly (9,9′-di-n-octylfluorenyl-2,7-diyl) (PFO) with each poly[2-methoxy-5-(2-ethylhexyloxy)-1,4-phenylenevinylene] (MEH-PPV) and poly[2-methoxy-5-(3,7-dimethyl-octyloxy)-1,4-phenylenevinylene] end-capped with Dimethyl phenyl (MDMO-PPV–DMP) by a systematic doping strategy was achieved. Both binary hybrid thin films of PFO/MEH-PPV and PFO/MDMO-PPV–DMP with various weight ratios were prepared via solution blending method prior to spin coating onto the glass substrates. The conjugation length of the PFO was tuned upon addition of acceptors (MEH-PPV or MDMO-PPV–DMP), as proved from shifting the emission and absorption peaks of the binary hybrids toward the acceptor in addition to enhancing the acceptor emission and reducing the absorbance of the PFO. Förster resonance energy transfer (FRET) is more efficient in the binary hybrid of PFO/MDMO-PPV–DMP than in the PFO/MEH-PPV. The efficient FRET in both hybrid thin films played the major role for controlling their emission and producing white emission from optimum ratio of both binary hybrids. Moreover, the tuning of the emission color can be attributed to the cascade of energy transfer from PFO to MEH-PPV, and then to MDMO-PPV–DMP.

## 1. Introduction

Organic-based materials for electronic applications, such as organic light-emitting diodes (OLEDs), have contributed significant advances in the field of polymer emitting devices. OLEDs gather several striking features, such as being low-cost; being easy to assemble by using wet methods; having a low power intake, owing to low conductivity of the organic materials; and having the ability to perform well in flat panel displays [1,2]. As white light is generally acquired by combining three principal colors (blue, green and red), two or more emitting constituents are piled in multilayer configuration [3,4,5,6] or assorted within a distinct layer by mixing or doping [7,8,9,10]. The latter methodology is favored, as the white OLEDs can be designed with a simpler configuration and a convenient approach escaping the vacuum coating (e.g., solution dispensation) for cost-effective white electroluminescent components.

The emission efficacy of OLEDs can be adjusted by matching the electron and hole inoculation through the combination of two dissimilar polymers with different electrical properties [11]. Further, by shifting the wavelength of the fluorescence emission from the absorption peak of the leading polymer, the optical properties of a polymer composite can be enhanced in relation to the reduced optical loss. The wavelength shift is attained by a non-radiative Förster-type energy transfer from the excited state of the donor polymer to that of the acceptor polymer [11]. The principle of a Förster-type energy transfer is a spectral overlay between donor emission and acceptor absorption, distance between donor and acceptor, positioning of the dipoles of acceptor and donor molecules and the prevailing medium [12,13]. The Förster-type radiative energy transfer (FRET) arises due to dipole–dipole interaction, in which the separation between donor and acceptor must be <100 Å [14,15]. To estimate the Förster radius, three different techniques have been considered: a direct measurement of the energy transfer rate [16], photoluminescence (PL) quantum efficiency [17] and spectral overlap [18]. In all the techniques, it is essential to approximate the polymer molecules to a hard sphere so that the concentration of a particular composite can be associated to the intermolecular distance of the donor and the acceptor. In the steady-state photoluminescence measurements, the energy transfer in most composites was observed to be a two-step process comprising exciton migration in the donor, followed by a Förster-type energy transfer from the donor to the acceptor molecule [19,20]. The FRET technique was exploited in the present work to produce WOLED with improved outcome. FRET excitation in the blend demands good mixing of the two materials and good spectral overlap between acceptor absorption and donor emission [21]. Blending of both donors and acceptors for energy transfer can significantly reduce the concentration quenching of the excitons formed, enhancing the device performance [22,23,24].

In the present work, we used the ternary blend of poly [9,9′-di-n-octylfluorenyl-2,7-diyl] (synonym: PFO), poly [2methoxy-5-(2-ethyl-hexyloxy)-1,4-phenylenevinylene] (synonym: MEH-PPV) and poly[2-methoxy-5-(3,7-dimethyl-octyloxy)-1,4-phenylenevinylene] end capped with Dimethylphenyl (synonym: MDMO-PPV–DMP) to achieve cascaded energy transfer for tuning emission colors and improving device performance. Three different emission components of PFO/MEH-PPV/MDMO-PPV–DMP were miscible with each other. Furthermore, the donor emission spectrum (PFO) and the acceptor absorption spectrum (MDMO-PPV–DMP or MEH-PPV) overlapped significantly. The main component of PFO acted as a diluent, matrix and excitation energy donor for the ternary blend, producing light with high efficiency. Therefore, when the ternary blend was excited near the absorption peak wavelength of PFO, light emission from MDMO-PPV–DMP and MEH-PPV was expected, suggesting the cascade energy transfer used for WOLEDs. As the research on the photophysical mechanism of ternary hybrid systems is rare, the current research is focused in detail on the study of controlling the emission spectrum of binary emitting polymer hybrids by a systematic doping strategy via FRET for white light emission.

## 2. Materials and Methods

**Figure 1:**
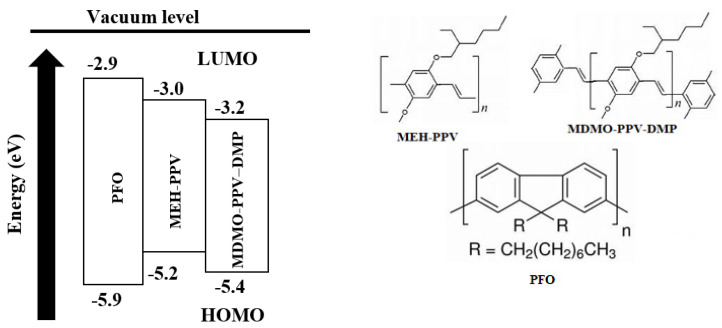
Energy-level diagram of the ternary polymers, and their chemical structures.

PFO and MEH-PPV, with an average molecular mass of 58,200 and 40,000 g/mol, respectively, were purchased from Sigma Aldrich (Saint Louis, MI, USA), whereas MDMO-PPV–DMP was purchased from American Dye Source, Inc. (Morgan Boulevard, QB, Canada) and used as received, without any purification. The structure of each polymer and its energy level are shown in Figure 1.

The two acceptors (MEH-PPV and MDMO-PPV–DMP) and the donor (PFO) were dissolved separately in toluene, and then various amounts of each acceptor solution, namely 0.1, 0.3, 0.5, 1.0, 2.0, 3.0, 5.0 and 10 wt.% (0.015, 0.045, 0.075, 0.15, 0.31, 0.46, 0.79 and 1.67 mg/mL) were mixed with a fixed concentration (15 mg/mL) of the donor. The binary hybrids with different weight ratios were spin-coated from the toluene solutions on clean glass substrates for absorption and emission spectra measurements. The optimum ratio of each binary hybrid was used to produce ternary hybrid. All thin films were controlled to be constant by taking 50 μL of each sample and then spin-coated at fixed 2000 rpm, with a deposition period of 20 s. All thin films were annealed at 120 °C for 10 min in a vacuum oven to remove the toluene. A surface profilometer (Dektak 150, Bruker, Billerica, MA, USA) was employed to measure the thickness of all thin films. The thickness of pristine PFO, MEH-PPV and MDMO-PPV–DMP was at around 110, 122 and 125 nm, respectively. For binary- and ternary-blend thin films, the thickness was slightly changed in the range of 113–120 nm. The absorption and emission spectra were collected by using a UV–Vis spectrometer (JASCO V-670, Cremella, Italia) and spectrofluorometer (JASCO FP-8200, Cremella, Italia), respectively. The emission data of each sample were employed to obtain the CIE coordinates, using the OriginLab program version 2019b (Northampton, MA, USA). All measurements were performed at ambient conditions.

## 3. Results

### 3.1. Optical Properties

**Figure 2:**
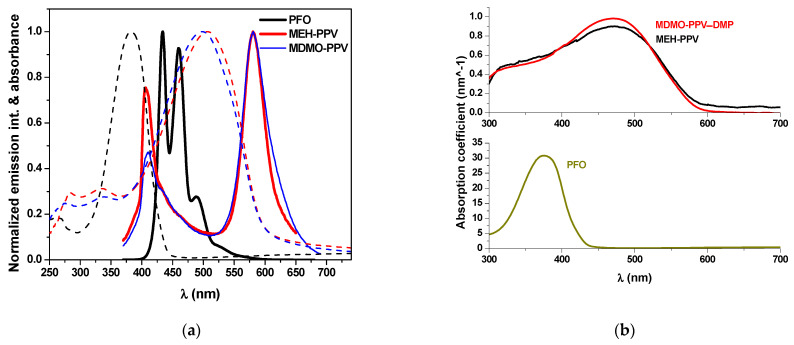
(**a**) Normalized absorbance (dash curves) and emission spectra (solid curves) of the pristine PFO (black colors), MEH-PPV (red colors) and MDMO-PPV–DMP (blue colors) thin films. (**b**) Absorption coefficient of pristine polymer thin films.

The normalized optical absorption and emission spectra of the pristine PFO, MEH-PPV and MDMO-PPV–DMP thin films are shown in Figure 2a. The extent of a polymer to absorb the light of a given wavelength can be represented by its absorption coefficient. The absorption coefficient of all pristine polymer thin films was indicated in Figure 2b. The lowest unoccupied molecular orbital (LUMO) and the highest occupied molecular orbital (HOMO) levels of the polymers, as well as the strong overlap of PFO emission with both absorption of MEH-PPV and MDMO-PPV–DMP, meet the necessary conditions of Förster-type energy transfer.

**Figure 3:**
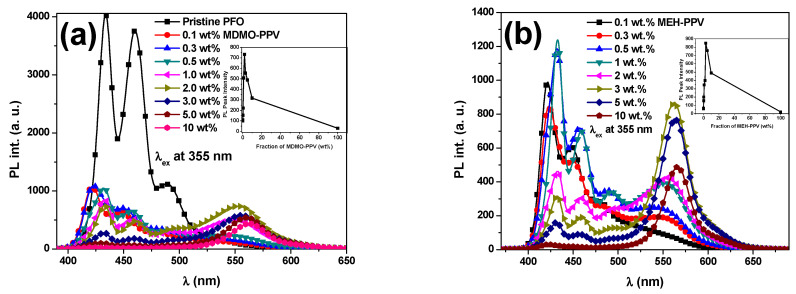
Emission intensity of the hybrids of PFO with different weight ratios of (**a**) MDMO-PPV–DMP and (**b**) MEH-PPV. All emission intensities were reduced ~20 times by using specific filter to avoid the saturation during the collection spectra. Insets in (**a**,**b**) are PL peak intensity of the hybrids measured at 550 and 565 nm as a function of the fraction of MDMO-PPV–DMP and MEH-PPV in weight percent ratio, respectively.

**Figure 4:**
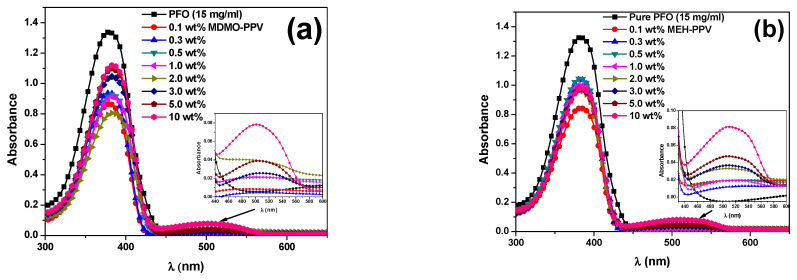
Absorbance of the hybrids of PFO with different weight ratios of (**a**) MDMO-PPV–DMP and (**b**) MEH-PPV. Insets are absorbance of each binary hybrid in the range of 440–600 nm.

It can be expected that the energy transfer from PFO to MEH-PPV and from PFO to MDMO-PPV–DMP will vary by varying the composition of each binary hybrid thin film. When the hybrid thin films containing the acceptor (MEH-PPV or MDMO-PPV–DMP) with a small fraction were excited at the absorption peak wavelength (355 nm) of the absorbing donor (PFO), the emission peak wavelength of the hybrid shifted towards that of acceptor and its intensity dramatically enhanced with increasing content of acceptor as shown in Figure 3. Moreover, Figure 4 shows that the absorption peak wavelength of the donor (~380 nm) was slightly red shifted and its absorbance dramatically decreased upon addition various content of the acceptor (MEH-PPV or MDMO-PPV–DMP) in the binary hybrids, which may lead to tune the conjugation length of the donor [24,25]. Additionally, new absorption peaks centered at 500 and 510 nm in the PFO/MDMO-PPV–DMP and PFO/MEH-PPV binary hybrids, respectively, were enhanced upon increments of the acceptor content, implying the possibility of dimer formation in the binary hybrids [14,26].

**Figure 5:**
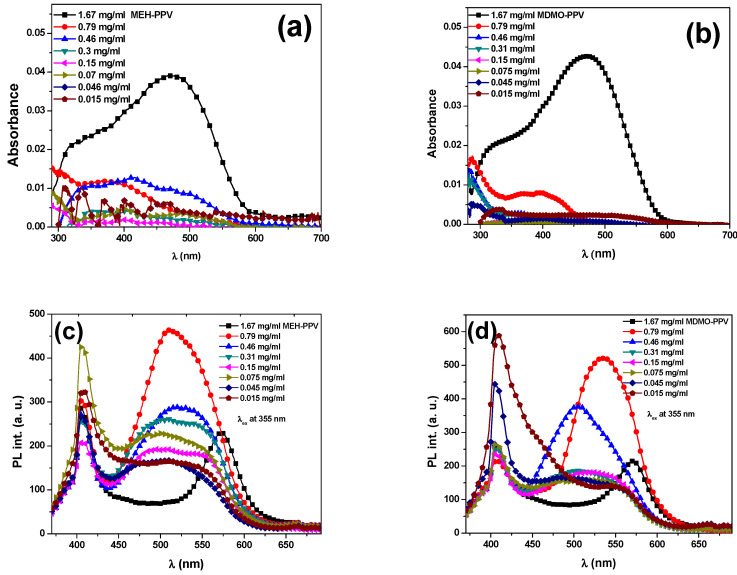
Absorption spectra at various concentrations of (**a**) pristine MEH-PPV and (**b**) pristine MDMO-PPV–DMP, and (**c**,**d**) their fluorescence spectra, respectively, with excitation wavelength of 355 nm.

The absorption and fluorescence spectra of pristine MEH-PPV and MDMO-PPV–DMP (at concentrations equivalent to the weight ratios used in the binary hybrids) are presented in Figure 5. The very weak emission intensities of these acceptors’ concentrations, compared with their corresponding emission intensities in the binary hybrids, give evidence for the efficient Förster-type energy transfer from PFO to both MEH-PPV and MDMO-PPV–DMP.

It is very interesting to note that the hybrid containing only 2.0 wt.% of acceptor in the binary hybrids of PFO/MDMO-PPV–DMP or PFO/MEH-PPV gives an emission spectrum that is the average of the individual spectra of PFO and MEH-PPV or MDMO-PPV–DMP when excited at the absorption peak wavelength of donor. The hybrids with more than 5 wt.% in each binary hybrid demonstrated an emission spectrum similar to that of pure acceptor, although a reduced full width at half maximum (FWHM) was observed. The peak of PL intensity at 550 nm of the binary PFO/MDMO-PPV–DMP blend (inset Figure 3a) was improved ~8 and 25 times compared with that in the pure PFO (Figure 3a) and pure MDMO-PPV–DMP (Figure 5d), respectively, whereas the peak of PL intensity at 565 nm of the binary PFO/MEH-PPV hybrid (inset Figure 3b) was improved ~12 and 42 times compared with that in the pure PFO (Figure 3a) and pure MEH-PPV (Figure 5c), respectively. Therefore, an efficient energy transfer from PFO to each acceptor can be confirmed. At a high content of acceptors (≥5 wt.% for MEH-PPV and ≥3 wt.% for MDMO-PPV–DMP), significant quenching can be observed instead of enhancement in the corresponding emission peak intensity of the acceptors. These quenching can be attributed to the heterodimerics (exciplexes) formation at a high acceptor content [27]. On the other hand, the quenching can also be attributed to the active self-quenching in the hybrids, resulting from the formation of homodimers (excimers) between monomers of the acceptor [28] that are then converted to heat.

An interesting phenomenon was detected at 10 wt.% of each acceptor (MEH-PPV or MDMO-PPV–DMP), where the PFO (donor) emission was simultaneously quenched with acceptor emission. This complete quenching of donor emission means that the energy transfer was completed at this acceptor ratio. Moreover, the reduction of the acceptor emission means that the energy transfer from the donor to the significant number of acceptor monomers was converted into heat rather than being emitted in the form of fluorescence, where some of the acceptor monomers act as dark quenchers without any fluorescence [27].

**Figure 6:**
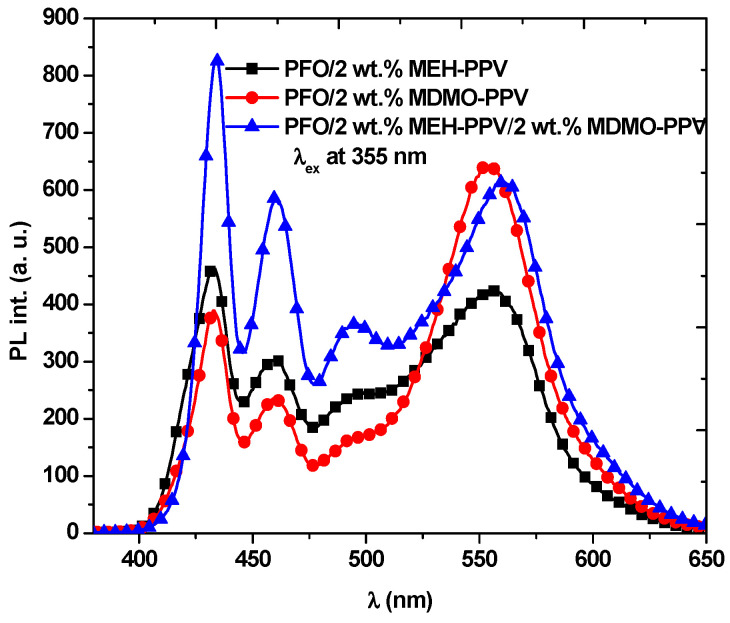
PL spectra of binary and ternary hybrids with the optimum ratios.

To avoid complete emission quenching, in particular from the donor, and to achieve white emission from the binary hybrids, it is of primary importance to precisely balance the delicate ratio between the donor and acceptors [29]. This ratio was obtained for the both binary blends with 2.0 wt.% of acceptor, as shown above in Figure 3. So, with this ratio of each acceptor, it can be expected to produce a PFO/MDMO-PPV–DMP/MEH-PPV ternary hybrid with white emission. To confirm this expectation, the PL spectra of each PFO/2 wt.% MDMO-PPV–DMP and PFO/2 wt.% MEH-PPV binary hybrids and their ternary hybrid were collected and are presented in Figure 6. The PL spectrum of the ternary hybrid with these desired ratios of the acceptors confirmed the production of white emission.

**Figure 7:**
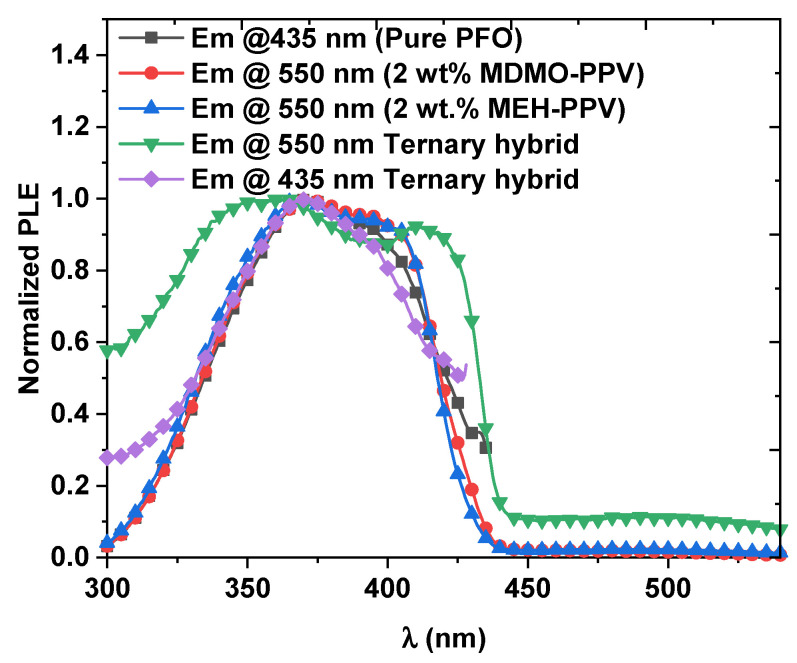
Normalized PLE spectra of pure PFO and the optimal ratios of binary and ternary hybrids at emission wavelengths of 435 and 550 nm.

Moreover, when the ternary polymers are molecularly intermixed within the Förster radius, the cascade energy transfer from PFO to MEH-PPV, and then to MDMO-PPV–DMP, can be expected. The cascade energy transfer can be confirmed by measuring the photoluminescence excitation (PLE) spectra of the ternary blend (Figure 7). When the emission at 550 nm was observed while changing the excitation wavelength, the PLE spectra were very similar to that of pure PFO thin film, which indicates that the emission from MEH-PPV mainly creates from the excitation of PFO. Furthermore, to confirm the energy transfer from PFO to MDMO-PPV–DMP in the ternary hybrid, the PLE spectrum at emission wavelength of 435 nm was measured. It is also very similar to the PLE spectrum of PFO. Therefore, in the order of PFO>MDMO-PPV–DMP>MEH-PPV, the cascade energy transfer is a highly convenient process. Meanwhile, a part of the PFO excitation energy is transferred directly to MEH-PPV because there is a part of spectral overlap between the MEH-PPV absorption and the PFO emission.

### 3.2. Energy Transfer Parameters

**Figure 8:**
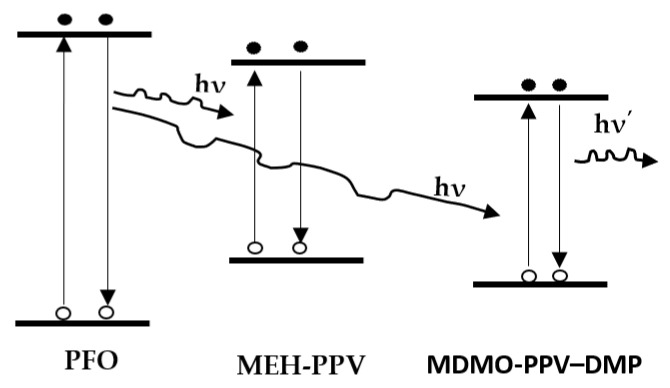
Schematic diagram of the FRET between PFO, MEH-PPV and MDMO-PPV–DMP.

The energy transfer is clearly observed in both PFO/MEH-PPV and PFO/MDMO-PPV–DMP hybrids when the MEH-PPV and MDMO-PPV–DMP content exceeded 0.1 wt.%, as shown in Figure 3. The possibility of energy transfer (Förster type) from PFO to each MEH-PPV and MDMO-PPV–DMP could be proved by (i) the strong overlap (Figure 2a) between the emission spectrum of pristine PFO and the absorption spectrum of each pristine MEH-PPV and MDMO-PPV–DMP, (ii) the strong reduction in the emission intensity of PFO with increment of each MEH-PPV and MDMO-PPV–DMP (Figure 3) and (iii) the significant improvement of the emission intensity of each MEH-PPV and MDMO-PPV–DMP. The schematic diagram of the FRET processes between these polymers is illustrated in Figure 8.

Numerous parameters of the energy transfer mechanism are evaluated in this section.

The quantum yield (ϕ_DA_) and lifetime (τ_DA_) values of the donor in both binary hybrid thin films were estimated by using the emission intensity of the donor in the absence (I_D_) and presence (I_DA_) of each acceptor, where in homogeneous dynamic quenching [15,30]:

$$\frac{I_{D}}{I_{\mathrm{DA}}}=\frac{\tau_{D}}{\tau_{\mathrm{DA}}}=\frac{\phi_{D}}{\phi_{\mathrm{DA}}}$$

**Figure 9:**
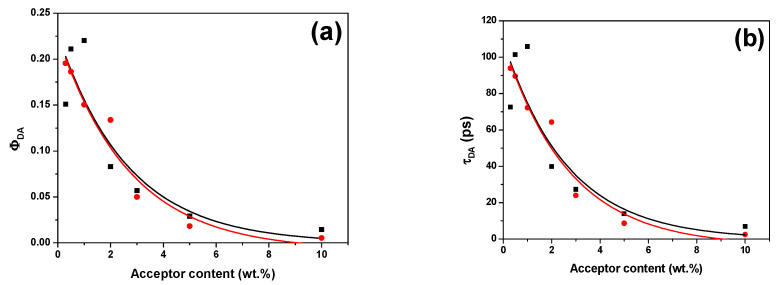
(**a**) Quantum yield (Φ_DA_) and (**b**) lifetime (τ_DA_) at various content of MEH-PPV (black color) and MDMO-PPV–DMP (red color). Solid curves refer to fitting data.

The reduction in values of both ϕ_DA_ and τ_DA_ with addition of each MEH-PPV and MDMO-PPV–DMP, as shown in Figure 9, suggested the possibility of radiative energy transfer. Furthermore, the shorter ϕ_DA_ and τ_DA_ values compared to those of the pristine PFO thin film (ϕ_D_ = 0.72 and τ_D_ = 346 ps [31]) provided evidence of the efficient energy transfer from PFO to each MEH-PPV and MDMO-PPV–DMP.

**Figure 10:**
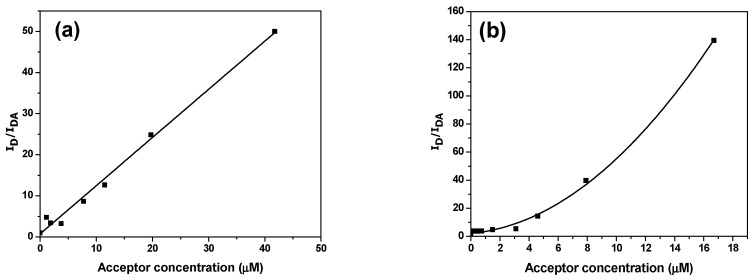
Stern–Volmer plots for emission quenching of PFO by various weight ratios of (**a**) MEH-PPV and (**b**) MDMO-PPV–DMP. Solid curves refer to the fitting data.

The linear Stern–Volmer plot (Figure 10a) confirms the homogeneity of the dynamic quenching of PFO by MEH-PPV, whereas the non-linear plot (Figure 10b) confirms that both types quenching, namely static and dynamic quenching, of PFO by MDMO-PPV–DMP can occur [15,32]. By fitting data of Figure 10 by using the OriginLab program and comparing them with the following Stern–Volmer equation, the Stern–Volmer constant (*k_app_*) can be estimated:

$$\frac{I_{D}}{I_{\mathrm{DA}}}=1+k_{app}\left[A\right],$$

where $k_{app}$ equals to slope of the linear fitting in Figure 10a and is equal to (*k*_S_ + *k*_D_) + *k*_S_ *k*_D_[*A*] in the non-linear fitting in Figure 10b [15]. *k*_S_ and *k*_D_ are static and dynamic quenching constants, respectively, and [*A*] is the acceptor concentration. According to Figure 10a, $k_{app}=1.173{\;\mu M}^{-1}$, whereas, from the fitting data of Figure 10b, we see that *k*_S_ = 0.056 µM^−1^ and *k*_D_ = 0.38 µM^−1^. These indicate that 50% of the fluorescence was quenched for the MEH-PPV and MDMO-PPV–DMP concentrations of 0.85 and 17.86 μM, respectively.

In order to determine the type of energy transfer between the monomers of PFO and each of MEH-PPV and MDMO-PPV–DMP, the critical transfer distance (R_0_) in Å was calculated by the following formula:

$$R_{o}^{6}=8.79\times 10^{-5}\left(\beta^{2}n^{-4}\phi_{D}\right)\int^{\;}F_{D}\left(\lambda \right)\varepsilon_{A}\left(\lambda \right)\;\lambda^{4}d\lambda =8.79\times 10^{-5}\left(\beta^{2}n^{-4}\phi_{D}\right)J\left(\lambda \right),$$

where J(λ) in units of M^−1^ cm^−1^ nm^4^, “*n*” is the solvent refractive index, *β*^2^ = 2/3 is the orientation factor for isotropic media, ε_A_(λ) is the molar decadic extinction coefficient of the acceptor in function of wavelength (λ) and *F_D_* (λ) is the normalized emission of the donor. The values of *J*(λ) and R_o_, as tabulated in Table 1, confirmed the dominant Förster type of energy transfer between the PFO monomers and each monomer of MEH-PPV and MDMO-PPV–DMP, where this type is typically effective in the range of 10–100 Å [33,34].

**Figure 11:**
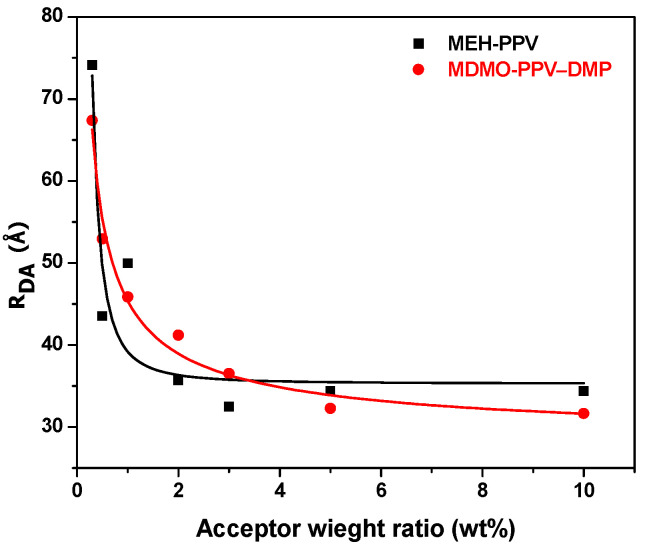
Distance (R_DA_) between the monomers of the donor/acceptor versus the acceptor content. Solid curves refer to the fitting data.

Based on Förster radio and emission intensities of the donor with (I_DA_) and without (I_D_) the acceptor, the distance (R_DA_) between PFO and each monomer of MEH-PPV and MDMO-PPV–DMP can be estimated as shown in Figure 11. As the content of each MEH-PPV and MDMO-PPV–DMP increased from 0.3 to 10 wt.%, the R_DA_ decreased from 74.1 to 36.9 Å and from 67.4 to 36.6 Å, respectively.

The energy-transfer rate (*k*_ET_) between a single pair of donor/acceptor in each binary hybrid, separated by an R_DA_, can be expressed in terms of R_0_ [12]:

$$k_{\mathrm{ET}}=\frac{1}{\tau_{D}}\;{\left(\frac{R_{0}}{R_{\mathrm{DA}}}\right)}^{6}$$

**Figure 12:**
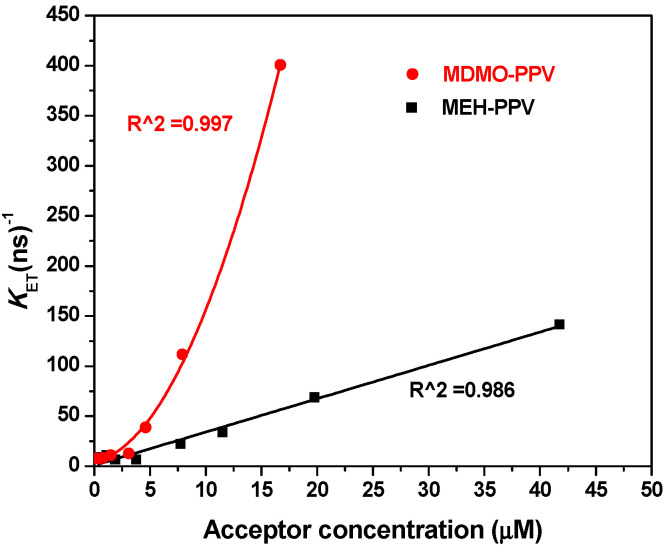
Energy-transfer rate (*k*_ET_) versus various weight ratios of MEH-PPV (black color) and MDMO-PPV–DMP (red color). Solid curves refer to the fitting data.

As shown in Figure 12, the *k*_ET_ values were significantly increased with the increasing the acceptor concentration, where they enhanced in the binary hybrid of PFO/MDMO-PPV–DMP greater than they did in PFO/MEH-PPV. This enhancement confirms the efficient energy transfer in both binary hybrids [15,30,35], and it was more efficient in the PFO/MDMO-PPV–DMP hybrid as compared to the PFO/MEH-PPV hybrid.

**Figure 13:**
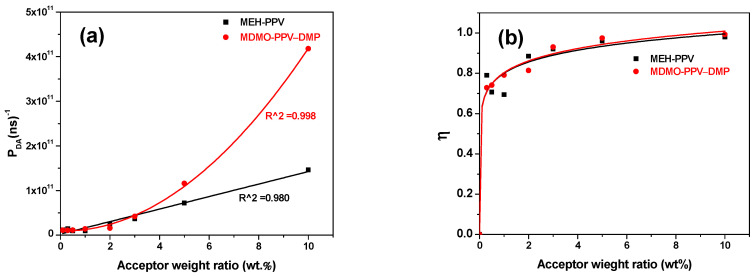
(**a**) Energy-transfer probability (P_DA_) and (**b**) energy-transfer efficiency (η) from PFO to each MEH-PPV and MDMO-PPV–DMP. Solid curves refer to the fitting data.

The relationship of the acceptor content with the probability (P_DA_) and efficiency (η) of the donor/acceptor energy transfer is illustrated in Figure 13, respectively. A gradual increase was observed for P_DA_ with the addition of the acceptor in both binary hybrids. The greater increase in values of P_DA_ in the PFO/MDMO-PPV–DMP hybrid can be attributed to the systematic decrease in the I_DA_ that was larger than that of PFO/MEH-PPV hybrid. On the other hand, we can also observe a systematic increase in the η until the acceptor content reached 2 wt.% and then remained fixed with a maximum value at 0.99 and 0.97 for binary hybrids of PFO/MDMO-PPV–DMP and PFO/MEH-PPV, respectively.

To terminate intermolecular transfer in the PFO, the MDMO-PPV–DMP and MEH-PPV concentrations should be much less than the critical concentration (A_o_), which is the acceptor concentration at which 76% of the energy was transferred [15]. Based on the average values of R_0_ [15,32], the A_o_ values of the MDMO-PPV–DMP and MEH-PPV were estimated as being ~1.73 and ~5.39 mM, respectively.

**Figure 14:**
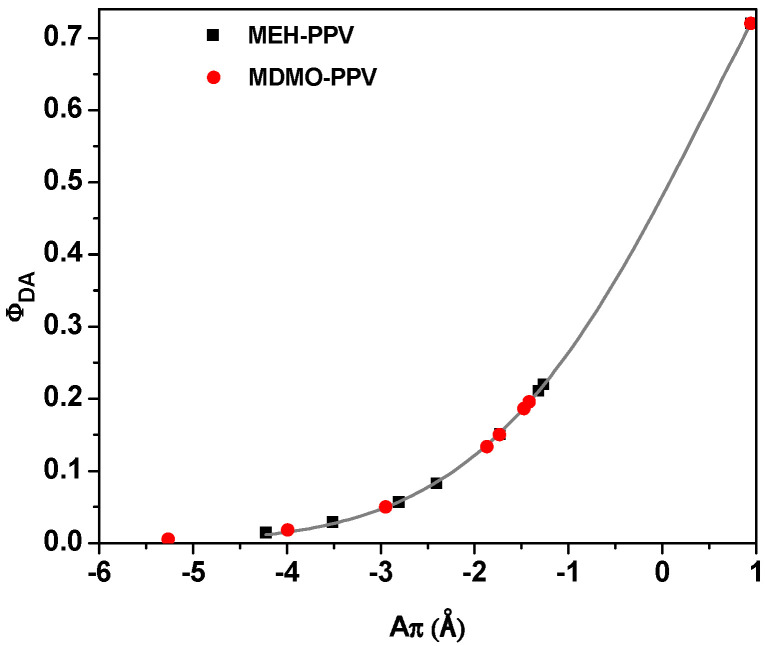
Fluorescence quantum yield (Φ_DA_) versus the conjugated length (A_π_). Solid curves refer to the fitting data.

On the other hand, based on the radiative (*k*_r_) and radiationless (*k*_nr_) rate constants [14,15], the conjugation length (A_π_) values in the excited singlet state for both binary hybrids were estimated. No significant difference was detected in the *k*_r_ value (~2.08 ns^−1^) with addition of MEH-PPV or MDMO-PPV–DMP, whereas the *k*_nr_ values were dramatically improved (Table 1). Consequently, the A_π_ of the PFO was decreased by increasing the acceptor in the PFO/MDMO-PPV–DMP hybrid more than that in the PFO/MEH-PPV hybrid. The exponential relationship between A_π_ and Φ_DA_ (Figure 14) confirmed that the addition of the MDMO-PPV–DMP or MEH-PPV produces binary hybrids with highly fluorescent. Moreover, an approximately linear portion can be observed when Φ_DA_ exceeded 0.2, with a zero value of A_π_ at Φ_DA_ ~0.5, which is consistent with the theoretical results [15,26].

### 3.3. Tuning the Emission Colors via FRET

**Figure 15:**
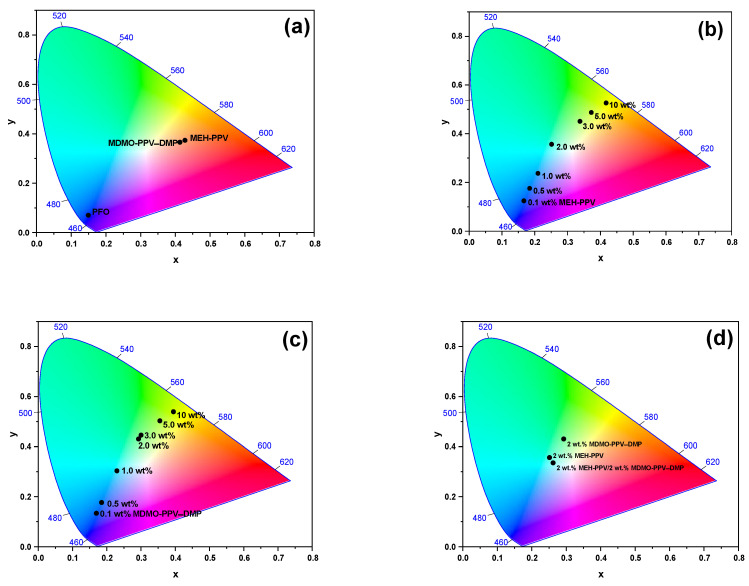
CIE coordinates of (**a**) pristine conjugated polymers, (**b**) PFO/MEH-PPV binary hybrids and (**c**) PFO/MDMO-PPV–DMP binary hybrids; and (**d**) optimum ratios of binary hybrids of PFO/MEH-PPV and PFO/MDMO-PPV–DMP and ternary hybrid of PFO/MEH-PPV/MDMO-PPV–P.

Figure 15 shows the CIE coordinates on the color chart for all pristine conjugated polymers, binary hybrids and ternary hybrid thin films. The CIE coordinates of pristine PFO (15 mg/mL), MEH-PPV (0.5 mg/mL) and MDMO-PPV–DMP (0.5 mg/mL) are (0.15, 0.07), (0.43, 0.37) and (0.41, 0.36), respectively, as presented in Figure 15a. Moreover, Figure 15b,c illustrated how the content of each MEH-PPV and MDMO-PPV–DMP, respectively, can be tuned the color emission of each binary hybrid via FRET mechanism. As the content of each MEH-PPV and MDMO-PPV–DMP in their binary hybrids increased, the CIE coordinates gradually shifted towards the white region before ended at the yellow region in the color chart. The shifting of CIE coordinates confirmed the efficient of FRET in the binary hybrids, as proved in the section above.

To obtain white emission, 2 wt.% of each acceptor MEH-PPV and MDMO-PPV–DMP was incorporated into the donor PFO. As a result, white emission (CIE coordinates: x = 0.26, y = 0.33) from the ternary hybrid was observed as demonstrated in Figure 15d. The tuning color can be attributed from the cascade of energy transfer from PFO to MEH-PPV, and then to MDMO-PPV–DMP, as expected later.

## 4. Conclusions

Tuning color emission of the hybrid thin films and producing white emission were achieved by a systematic doping strategy via FRET. A systematic increase in the FRET efficiency was observed with increasing the acceptor content until it reached 2 wt.% and then remained fixed with a maximum value at 0.99 and 0.97 for binary hybrids of PFO/MDMO-PPV–DMP and PFO/MEH-PPV, respectively. The quenching type of emission in the PFO/MEH-PPV binary hybrid was dynamic, while it was both static and dynamic in PFO/MDMO-PPV–DMP. To terminate the intermolecular transfer in the PFO, the concentrations of MDMO-PPV–DMP and MEH-PPV should be much less than ~1.73 and ~5.39 mM, respectively. The white emission (CIE coordinates: x = 0.26, y = 0.33) from the ternary hybrid was produced at the optimal ratio of PFO/2 wt.% MEH-PPV/2 wt.% MDMO-PPV–DMP.

## Figures and Tables

**Table 1:** Parameters of energy transfer from PFO to both MEH-PPV and MDMO-PPV–DMP.

In PFO/MEH-PPV Binary Hybrid					In PFO/MDMO-PPV–DMP Binary Hybrid			
Acceptor Content (wt.%)	MEH-PPV Conc. (mg/mL)	*K*_nr_ (ns)^−1^	*J*(λ) × 10^15^ (M^−1^.cm^−1^.nm^4^)	R_0_ (Å)	MDMO-PPV–DMP Conc. (mg/mL)	*K*_nr_ (ns)^−1^	*J*(λ) × 10^15^ (M^−1^.cm^−1^.nm^4^)	R_0_ (Å)
0.1	0.375	9.76	284	111	0.15	8.843	329	113
0.3	1.125	11.69	94.6	92.5	0.45	8.564	38.0	79.4
0.5	1.875	7.77	0.674	40.6	0.75	9.082	9.5	63.1
1.0	3.75	7.36	5.34	57.3	1.5	11.76	5.31	57.2
2.0	7.75	23.02	0.128	30.8	3.1	13.47	3.24	52.7
3.0	11.5	34.48	0.355	36.5	4.6	39.55	4.80	56.3
5.0	19.75	69.69	5.97	58.4	7.9	112.6	6.6	59.3
10	41.75	142.3	18.8	70.6	16.7	401.3	50.7	83.4

## Data Availability

The data presented in this study are available on request from the corresponding author.
